# Common-sense approaches to sharing tabular data alongside publication

**DOI:** 10.1016/j.patter.2021.100368

**Published:** 2021-12-10

**Authors:** Nicholas J. Tierney, Karthik Ram

**Affiliations:** 1 Monash University, Department of Econometrics and Business Statistics, Melbourne, Australia; 2 Australian Centre of Excellence for Mathematical and Statistical Frontiers (ACEMS), Melbourne, Australia; 3 Telethon Kids Institute, Perth Children's Hospital, Perth, Australia; 4 Berkeley Institute for Data Science, University of California, Berkeley, USA

**Keywords:** DSML 4: Production: Data science output is validated, understood, and regularly used for multiple domains/platforms

## Abstract

Numerous arguments strongly support the practice of open science, which offers several societal and individual benefits. For individual researchers, sharing research artifacts such as data can increase trust and transparency, improve the reproducibility of one's own work, and catalyze new collaborations. Despite a general appreciation of the benefits of data sharing, research data are often only available to the original investigators. For data that are shared, lack of useful metadata and documentation make them challenging to reuse. In this paper, we argue that a lack of incentives and infrastructure for making data useful is the biggest barrier to creating a culture of widespread data sharing. We compare data with code, examine computational environments in the context of their ability to facilitate the reproducibility of research, provide some practical guidance on how one can improve the chances of their data being reusable, and partially bridge the incentive gap. While previous papers have focused on describing ideal best practices for data and code, we focus on common-sense ideas for sharing tabular data for a target audience of academics working in data science adjacent fields who are about to submit for publication.

## The bigger picture

Without data, there is no science. Science needs to be reproducible so we can trust the results and progress as a field. Although academia generally appreciates the benefits of data sharing, research data are usually only available to the original researchers. If data are shared, they often lack the documentation to make them easy to reuse. There is not a strong culture of data sharing, and we believe this is due to a lack of incentives and infrastructure for making data useful. While past papers focus on general practices for sharing data and code, we focus on a specific audience: academics working in data science adjacent fields who are about to submit for publication. We provide immediately usable guidelines to share tabular data alongside research that a researcher could pick up and use today. In the future, we hope the culture around data sharing will change and be rewarded in science. A new normal is needed where data are submitted with every research publication, and datasets are easily discovered, shared, and extended in other analyses.

## Teaser

Despite a general appreciation of the benefits of data sharing, research data are often only available to the original investigators. We argue the lack of incentives and infrastructure for making data useful are the biggest barriers to widespread data sharing. We compare data sharing with code sharing, in the context of facilitating reproducible research. We provide a common-sense guide for sharing tabular data alongside research. Our target audience is academics in data science adjacent fields who are about to submit for publication.

## Introduction

“Data! data! data!” he cried impatiently. “I can't make bricks without clay.” – Sherlock Holmes (“The Adventure of the Copper Beeches” by Sir Arthur Conan Doyle)

The idea of open and transparent science dates back to the founding principles of The Royal Society of London in 1660. The movement, which has seen shifting support over the years, has enjoyed growing popularity over the past decade,^1^^,^^2^ primarily among early career researchers.^3^ A key motivation for practicing openness is the notion that sharing all research artifacts will allow others to reproduce and verify results, thereby improving trust in and verification of those findings. Jon Clarebout famously wrote in 1992 that reproducibility in computationally oriented fields had become so trivial that nonexperts could do the task. Clarebout's optimistic vision was a little ahead of its time, as the tools and technologies available in the early 1990s for literate programming presented too high a barrier for most researchers to adopt without incurring a significant loss in productivity.^4^

Meaningful progress on most computational reproducibility, the ability of an independent researcher to recreate figures, tables, and key findings, became more of a practical reality in the late 2000s as new tools and services came into existence. Scientists started sharing more code and software, partly due to the rapid increase in training opportunities made possible by efforts like The Carpentries, combined with the growing adoption of GitHub by scientists.^5^^,^^6^ The bigger driver for this is more likely the growing popularity of data science as a discipline distinct from statistics.^7^ This rapid growth in data science has been catalyzed by a Cambrianesque explosion of open-source software tools. Programming languages such as Python, R, and Julia have helped scientists implement and share new methods for working with data. Each of these languages enjoys the support of thriving communities of researchers and software developers who contribute many of the building blocks that make them popular. Modern scientists now have access to many interactive computational frameworks (e.g., Jupyter, RMarkdown, Stencila, Observable) that can interface with kernels from dozens of programming languages. The barriers to verification are now low enough that reproducibility is sometimes a click away, free from the misery of software installation.^8^

Notebooks, however, are an incomplete solution for computational reproducibility. For a piece of computational research to be minimally reproducible, it requires three distinct elements: (1) code, (2) computing environment, and (3) data. There has been tremendous progress on the first two, despite some limitations of existing tools and practices. While it took nearly two decades for Clarebout's vision to become reality, improvements in technology and motivations have played a strong role in the culture change. Sharing code was not easy at first,^4^ but became a social and normative part of academic culture. The next stage of this evolution, turning code into software, also became popular with increasing training opportunities,^9^ tooling, and recognition of code as a scholarly contribution.^10^ Getting researchers to share usable data has been more of a struggle.

**Figure 1:**
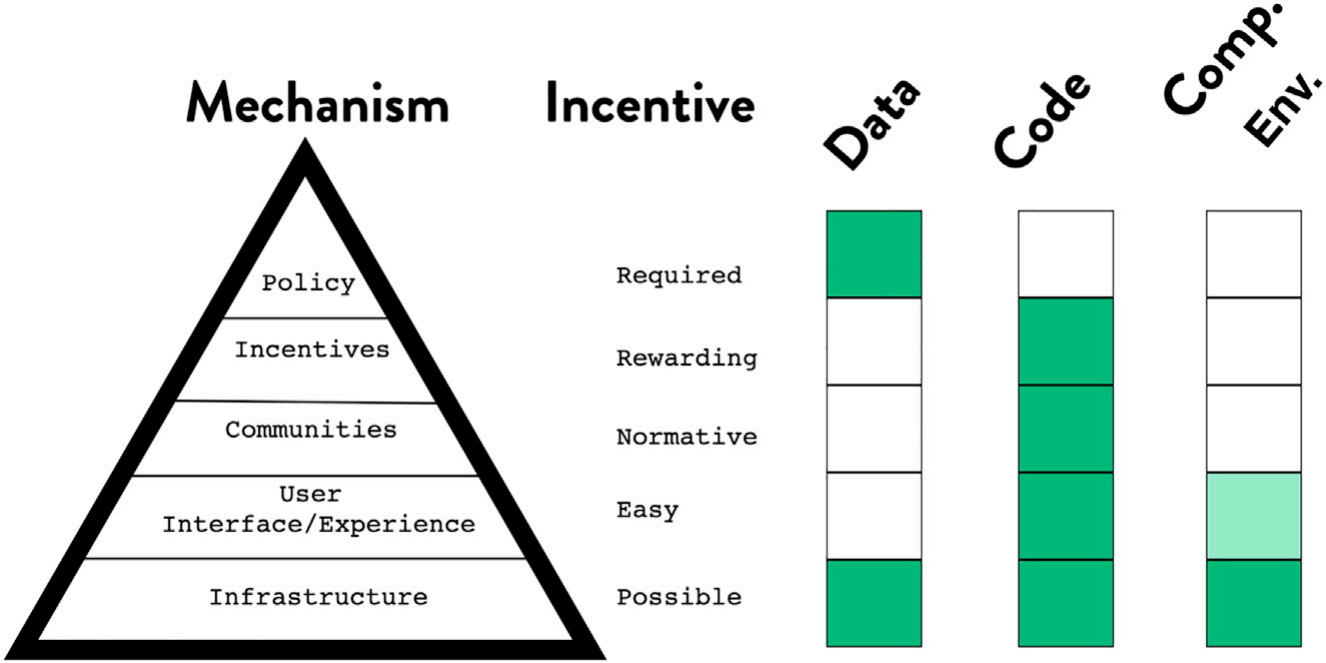
The mechanisms for behavior change, the incentives, and our assessment of where the elements of data, code, and computational environment rank in terms of completing these aspects. We note that data are often required, but the preceding steps are not, in contrast to code, which has no policy.

Nosek^11^ describes the strategies for culture change, especially when the status quo is too entrenched. For change to occur, more than just motivation is needed: Skills, tools, and invectives are critical. In a five-step process, Nosek describes how achieving lasting culture change requires us to show proof of concept that an activity is possible, improve the infrastructure to make it easy, and then increase awareness to make it normative. Once there is a critical mass, ways must be found to reward those who put it into practice. Once change has become normative and is widely practiced, it is possible to require it of everyone (Figure 1). This framework provides a useful lens through which to examine adoption of code, computational environments, and data for reproducibility. With code, we have slowly progressed through these stages. While few journals require that authors share code, the provision of venues within which code can be shared, along with the necessary training, has paved the way for incentives and credit. All of the necessary foundations to make code sharing required are in place. For computational environments, Docker makes it possible to share them in most common use cases.^12^ However, running them still requires expertise and time on the part of reviewers. More recent open services, like Binder,^8^ make it possible to run notebooks atop Docker images, but the technology is still new and experiencing growing pains. There is still a long way to go before sharing computational environments in academia becomes mainstream.

Finally, for data, we have implemented these steps out of order. The FAIR data principles, introduced in 2016 with good intentions,^13^ describe the desired attributes for research data: that they be findable (good metadata), accessible (deposited somewhere persistent), interoperable (easily combined with other datasets), and reusable. We, as a community, started out by making data required before demonstrating how this would directly benefit those who share data. Researchers are asked to deposit their data in one of many institutional or commercial repositories. Many journals even require it as a condition for publication via policy. However, the cost of preparing data for reuse remains high, and career incentives to do so are almost nonexistent. Until we make data reuse easier and more rewarding, no level of policy is going to improve the state of data sharing.

While there has been a growing trend of sharing data alongside publications, the status quo of data sharing in science is not good. In 2016, Rowhani-Farid et al.^14^ showed that seven of 157 (4.5%) sampled papers in the *British Medical Journal* shared data, and only 14% of articles claiming open sharing of data actually shared their data. Similarly, in 2018, Stodden et al.^15^ showed that only 36% of papers published in the journal *Science* shared data, despite the journal's data sharing policy. Similar findings have been made for PLOS and BMC journals.^16^ There are numerous examples of similar problems in other fields, such as psychology^17^ and biology.^18^ The reality is that while data sharing is increasing in many fields, such as ecology and genomics, the amount of data that can be reused is likely lower. To make shared data useful, we need to realign incentives for researchers.

Tools that can be used to prepare datasets and metadata (the infrastructure) and minimal sets of practices (guidelines) for reuse are sparse.^19^^,^^20^ Unlike code, datasets are far more diverse in terms of heterogeneity, size, and format. This makes them particularly challenging to standardize or easily “install” where the code is running. There have been some efforts to improve this process, such as gogetdata, the Dat Project, Frictionless Data,^21^ and the formation of a private company, Quilt.

Depositing data with rich metadata is laborious, making it a *pro forma* activity, outside of specialty repositories like Dryad that employ curators. Finally, the infrastructure to track and get credit for data does not yet exist. Datasets currently do not accrue citation credit since they are not tracked.^22^

## Improving the state of reusable data sharing

We believe a key audience could be better served by the current literature on data sharing: researchers who have finished data analysis on tabular data and are ready to submit for publication. Many papers provide rules and guidelines for sharing data, such as providing metadata, citations, licenses, and lists of data repositories to use. These papers provide general advice that ranges from ethical considerations raised by sharing data,^23^ to how to share many different data types (e.g., images, video),^24^ to tips such as “reward colleagues who share their data properly.”^25^ There is also specific advice, such as the importance of very clean and properly shaped data,^26^ and how to use open-source software with changes tracked.^27^ We walk a fine line between providing highly specific advice (e.g., how to specify null values) and overly general suggestions (e.g., sharing data is good). Our goal is to provide practical advice for researchers who are familiar with modern data science tools and who already develop their work, including research compendia.

As academic data scientists, we spend considerable time cleaning messy data and believe that preparing data for easy reuse can be a catalyst to improve the quality of data and pave the way for other incentives, such as community adoptions, which will then create the culture change needed to credit data in a way similar to the citations used to credit publications. In this paper, we describe a set of common-sense practices that researchers can adopt to make data more useful in the context of reproducibility. It should not be a huge burden to share data in a format that is readily usable by others. Similarly, this burden should be reduced by and for those who want to use another's data.

To make data more reusable, we need to make it easier to generate metadata, standardize good practices within and across communities, and make reusable data sharing a recognized, normalized part of research. Here we present a set of common-sense practices to facilitate this change. These include documentation (in the section Documentation [READMEs]), licensing (section Licenses), metadata (section Metadata), raw and analysis-ready data (section Raw data versus analysis-ready data), code (section Data cleaning scripts), avenues to share data (section Avenues to share data), and citation (section Citation: How you want your data to be cited). We also discuss considerations for sharing in packages (section Data as part of open-source packages) and sharing data of various sizes (section Considerations for data size).

We limit the scope of this paper to small to medium-sized tabular data, which includes a vast majority of use cases that researchers encounter day to day. Larger datasets with complex workflows (e.g., bioinformatics and medical imaging) face different kinds of challenges and accordingly deserve to be treated separately.

Assuming that the data have no privacy concerns (e.g., human participants, locations of critically endangered species), or that the act of sharing does not put the authors at a competitive disadvantage (data can be embargoed for reasonable periods of time), sharing data will always have a net positive benefit.

Our advice is not meant to be exhaustive, by any means, and is insufficient to fully bridge the gap, but we hope instead to provide a starting point.

### Documentation (READMEs)

Use a README file with your data project to communicate its purpose and contents. A README is often the first place users will look to learn more about a project. The README is a concise text file containing guidance on how to read and interpret the directory: the who, what, when, where, and how (https://betta.io/blog/2017/02/07/developer-experience-github-readmes/). For example, a README in a data repository might explain which directories contain data, tidy scripts, and also why data were collected. The README should be placed in the top level of the project, and there can be multiple README files in a project (e.g., in different directories). In the context of datasets, READMEs are particularly useful when there are no reliable standards. Although data repositories contain a large number of datasets associated with peer-reviewed papers, not all are usable (e.g., https://datadryad.org/stash/dataset/doi:10.5061/dryad.rn6k1). Some of the more recent submissions contain usage notes and a README, which considerably improve reuse potential (e.g., https://datadryad.org/stash/dataset/doi:10.5061/dryad.3xsj3txbz).

The date of use, associated paper, and versions of software should be recorded in the README, so users can, in the future, replicate similar computing environments or conditions. A more ideal approach would be to clearly describe the computing environment (e.g., in a Dockerfile) and packages used (e.g., requirements.txt).

### Licenses

Provide a license file with your data. A license for data establishes the rules for their use and whether they can be modified or shared. Without a license, or with a nonstandard one, these rules are unclear and can lead to issues with use, attribution, and citation. While there are a handful of licenses for data, we recommend using the CC0 license, which means that data owners waive all rights to the data, so they can be freely shared, copied, modified, and distributed, without permission or attribution, even for commercial purposes.

We recommend the CC0 as it provides simple rules for attribution, warranty, and use, and is very common. If the CC0 license is used in scientific publications, you need to cite the location from which you obtained the data as part of scientific practice. There might be some concern that it is possible to conduct fraud, but any such fraud is likely to be found out eventually if the original data are archived properly.

For more information on the benefits of using CC0, and why CCBY is not always appropriate, see https://osc.universityofcalifornia.edu/2016/09/cc-by-and-data-not-always-a-good-fit/. Short and full overviews of CC0 can be found at https://creativecommons.org/.

### Metadata

Include metadata with your data. There are three benefits to a dataset that includes metadata: (1) it provides context by describing variables to humans, (2) it avoids errors by describing and preserving variable types for machine readability, and (3) it makes data easier to find online by facilitating indexing.^28^ Three pieces of metadata should be considered: (1) variable names, (2) variable descriptions, and (3) unit text. These should be curated by a human. Metadata created should be clearly labeled in a folder or in files named METADATA.

Variable names should be short, descriptive names with no spaces or special characters, such as “job_position” or “date.” Variable description describes the measured variables (e.g., “university job position” or “date started”) and should also define variable codes and missing data. Unit text defines the type of variable recorded (e.g., string, date, numeric, integer) in order to avoid serious errors of misrepresented data (e.g., a column of a gene sequence being interpreted as a date).

A simple metadata file that describes the variable names, variable description, and unit text is a great starting point, and is sometimes known as a codebook. Metadata can be encoded into more formal plain text formats that follow a set standard, such as EML or SCHEMA-LD. A deep understanding of these formats is not required to use them. More details are available elsewhere.^19^^,^^29^, ^30^, ^31^

### Raw data versus analysis-ready data

Make both raw and analysis-ready data available. Raw data are the first format of data provided before any cleaning has taken place (e.g., data in some binary or proprietary format). They should be placed in their own folder. Data used in analysis should be provided, especially if they were computationally intense to derive. They should be in their own folder and, to maximize interoperability, recorded in a plain text (e.g., csv, tsv) and, if possible, in a binary format (e.g., .rds, .sav, .xlsx, Apache Parquet). The binary format allows for immediate reuse in the intended state, while the interoperability of plain text versions remains stable over time. Ideally, data should be in “tidy data” form, where variables are in columns, observations in rows, and each cell has only one value.^32^

### Data cleaning scripts

Share code used to transform data from their raw format into analysis-ready form. This should be kept in the same place as raw data. Ideally, this transformation should involve only scripted languages (e.g., R, Python, MATLAB), but any unscripted data preparation steps should be recorded in a plain text file. Authors should run the scripts in a clean environment to ensure that everything runs and that the code does not rely on other local artifacts, such as their own folder layout not available to others.

For more complex analyses that involve workflow management systems (see https://github.com/pditommaso/awesome-pipeline for a comprehensive list), a description of how to run the workflow should be included in the documentation, preferably in the README file. Depending on whether the workflow is GUI driven (e.g., Galaxy) or text driven (e.g., Nextflow, Snakemake), the workflow itself can be checked into the version control repository. As these communities work closely and use Common Workflow Language as a shared representation, these files can be widely used to recreate the steps involved in producing the final results.

### Avenues to share data

Data should be deposited into a relevant domain repository that generates an accession number such as a DOI (e.g., gbif for species data, or GenBank for genetics data). An exhaustive searchable list is available at re3data (https://www.re3data.org/) but provides no guidance to researchers. Data that do not fit appropriate domain repositories can be submitted to research data repositories Zenodo and Dryad, which are currently leading the way in best practices for software and data. In addition to minting DOIs, they also provide citation templates.

If using a research institute's library service to share data, ensure that a DOI is provided so it can be accurately identified and cited. Do not deposit data in local file storage that does not provide DOI or accession numbers.

Although it is fine to share data among collaborators using temporary cloud storage (like Dropbox or Google Drive), once the paper is public, the data need to be hosted somewhere persistent. Although a cloud-based storage approach is useful while iterating on a paper, it does not scale to a larger readership and is highly prone to disappearing (e.g., lack of permissions). Providing back-up data is particularly relevant in light of cases in which data are suspected of being tampered with.^33^^,^^34^ The use of raw data allows for issues to be identified; the absence of raw data in publications creates difficulties in verifying the validity of the data.

Journals provide data papers in a formal publication explaining data that are archived (e.g., *Ecology*, *Scientific Data*). This is a familiar avenue for researchers to receive credit and citation. Writing a data paper can be very useful for researchers, but journals that publish data papers lack guidelines on how to structure data for effective reuse and sharing. We recommend partnering with best practice data repositories that emphasize curation, like Dryad. This way the data will have long-term archiving and will also have professional curation to ensure that the published data are usable and have enough metadata to be understood. While writing a data paper can be very useful for researchers, journals that publish data papers lack guidelines on how to structure data for effective reuse and sharing. Data journals should routinely be partnering with best practice repositories to best support the underlying data.

### Citation: How you want your data to be cited

One critical advantage of depositing data in a trusted repository (see Avenues to share data) is that it comes with many benefits, such as preservation, indexing, discoverability, and, most importantly, citation with a persistent identifier. Authors should add a CITATION file when possible, but the real burden lies with publishers to ensure that these citations are marked up appropriately to allow for the building of open data metrics.^35^,^36^

When citing data, you should cite the preferred CITATION file in the first instance, or else a data publication (e.g., in *Scientific Data*, *Ecology*), and if none of those apply, cite the repository in which the data are deposited (e.g., a Zenodo repository) in the references section of related papers.

### Data as part of open-source packages

Data can be distributed alongside code using a format such as a package in R, Python, and Julia. A package or module structure provides an advantage, as it can be installed and loaded just like code. An obvious benefit of this approach is that it allows data to be immediately used in a programming environment without the additional steps of downloading, preprocessing, specifying column types, etc. But there are some limitations to this approach. First, packaging data inside a software package makes the data availability language-centric. Users who work in other languages are unlikely to download and export data out of a package. Second, data size can be a limitation for some repositories (CRAN, the R repository, for example, limits package sizes to 5 Mb, although there are workarounds). Other fields, such as bioinformatics, have a process of sharing data through repositories like Bioconductor, which can accommodate a larger data size. Third, there is the cost of authors and data curators/custodians understanding the intricacies of package development in order to share data. Nevertheless, the frictionless approach to sharing data in software packages makes reuse substantially faster than retrieving and correctly parsing files from a repository. However, we recommend the software approach only as an additive to proper data curation.

Packaging data in software and archiving in data repositories are not mutually exclusive activities. First, archive data in a long-term data repository (see Avenues to share data), before including it in a package. The act of packaging data in software can shave hours off the time required to prepare them for use. The canonical citation can be baked into the package's citation file.

### Considerations for data size

How data are shared can change depending on their size. Defining data size will always be somewhat difficult to do, since storage capacity and norms constantly change. We consider data sizes in reference to a moderately priced laptop, where small data fit on computer memory (RAM), medium data do not fit on RAM, and big data do not fit on the hard disk. No matter the size, data should be archived with an accession number or DOI.

Small data have more options for how they can be shared; you can bundle them with your analysis code (as in the section Raw data versus analysis-ready data) or in software (as in the section Data as part of open-source packages). For medium data, a general-purpose data repository (e.g., Zenodo) would be ideal for archiving. Sharing medium data might require them to be broken into separate chunks for reading (e.g., teaching-1.csv, teaching-2.csv), and scripts might need to be provided to read them. Large data will require liaising with services discussed in the section Avenues to share data, or making special arrangements with a local research institute library, to ensure that there is some way to gain access, and to maintain a DOI.

## Conclusion

Although data sharing is widely mandated by funders, journals, and academic societies, the amount of usable data remains appallingly low. The mere act of depositing data does not make them reusable or useful to a potential consumer. Although data citations do not currently affect promotion and tenure packets, there is some evidence that papers sharing usable data accrue more citations.^37^ Over time, the more useful we make the data, the more likely it becomes that incentive mechanisms will take hold.

The steps leading up to requiring data sharing are still vague and disincentivized. In comparison, although few journals require that code be submitted with publications, such a norm is more likely to be adopted if a foundation like the one we have described is established, with carrots rather than sticks. We have taught researchers how to write modular code via popular training initiatives such as The Carpentries. Commercial services like GitHub have made code sharing fun and productive. For more advanced researchers, data science languages have made it easier to package and disseminate code as reusable software. If we now require mandatory code sharing, it is quite likely that more of it will be useful than the data currently being shared. Researchers will need to be familiar with existing tools and approaches for sharing data. There might be potential for researchers to reinvent the wheel, for example by creating new platforms for data sharing, when services such as Zenodo and Dryad already exist, so it is worth mentioning that researchers should not reinvent new tools without an exhaustive search.

To make data sharing truly useful, we need to make the process easier, improve tools and training, and reward and incentivize researchers who engage in such practices. Only then can shared data truly be useful.
